# Supplementary material for: Complexome profiling of the Chlamydomonas psb28 mutant reveals TEF5 as an early PSII assembly factor
Source: Plant Cell. 2025 Mar 17;37(6):koaf055. doi: 10.1093/plcell/koaf055 (PMC12164590; doi:10.1093/plcell/koaf055)
Supplement: koaf055_Supplementary_Data [file koaf055_supplementary_data.zip › psb28_interactive.zip!/plots/intensity/qualityPlots/peptides/FAP303_Cre12.g528000.t1.2.html]

### Cre12.g528000.t1.2

Trivial name: FAP303  
SeqCoverage [median]: 15.60  
Mass: 44.20 kDa  
  
 

| Sequence | MissedCleavage | Mass [Da] | wtIntensity | muIntensity | Score |
| --- | --- | --- | --- | --- | --- |
| AVPLPTLTPSATFGK | 0 | 1498.84 | 1198.98 | 3420.93 | 68.36 |
| EGFQVLEPK | 0 | 1045.54 | 339.30 | 1789.45 | 60.59 |
| GPSAEEAIK | 0 | 900.46 | 0.00 | 468.91 | 105.14 |
| IFVVDPAK | 0 | 887.51 | 0.00 | 2131.63 | 67.98 |
| LIGSVSISDLR | 0 | 1158.66 | 1250.03 | 5373.36 | 96.33 |
| LLETMAGQGLHR | 0 | 1324.69 | 1685.08 | 5606.94 | 87.00 |
|  |  |  | 4473.4 | 18791.2 |  |
